# A simple saccadic reading test to assess ocular motor function in cerebellar ataxia

**DOI:** 10.1371/journal.pone.0203924

**Published:** 2018-11-07

**Authors:** Angela Jinsook Oh, Tiffany Chen, Mohammad Ali Shariati, Naz Jehangir, Thomas N. Hwang, Yaping Joyce Liao

**Affiliations:** 1 Department of Ophthalmology, Stanford University School of Medicine, Stanford, California, United States of America; 2 Department of Ophthalmology, Kaiser Permanente Redwood City Medical Center, Redwood City, California, United States of America; 3 Department of Neurology, Stanford University School of Medicine, Stanford, California, United States of America; Chinese Academy of Sciences, CHINA

## Abstract

Cerebellar ataxia is a neurological disorder due to dysfunction of the cerebellum that affects coordination of fine movement, gait, and balance. Although ataxic patients commonly exhibit abnormal eye movement and have difficulties with saccadic reading, quantification of ocular motor abilities during reading in the clinical setting is rarely done. In this study, we assess visual performance with simple reading tests that can be used in the clinical setting and performed video infrared oculography in 11 patients with hereditary or acquired cerebellar ataxia and 11 age-matched controls. We found that compared with controls, ataxic patients read significantly slower on regularly and irregularly spaced 120 single-digit number reading tasks (read aloud) (p = 0.02 for both) but not on a word reading task (read silently), although there was large variability on the word reading task. Among the 3 reading tasks, the regularly spaced number reading task had the greatest difference (44%) between ataxic patients and controls. Analysis of oculography revealed that ataxic patients had slower reading speeds on the regularly spaced number reading task because of significantly higher saccade and fixation counts, impairment of small amplitude progressive saccades as well as large amplitude, line-changing saccades, greater fixation dispersion, and irregularity of scan paths and staircase gaze patterns. Our findings show that infrared oculography remains the gold standard in assessment of ocular motor difficulties during reading in ataxic patients. In the absence of this capability in the clinical setting, a simple 120 regularly spaced single-digit saccadic number reading test, which most patients can perform in less than 2 minutes, can be a possible biomarker for ocular motor abilities necessary for reading.

## Introduction

Neurological issues like stroke, cancer, neurodegeneration, and genetic syndromes that affect the cerebellum can lead to cerebellar ataxia, which is a heterogeneous group of disorders characterized by loss of fine motor control or coordination, difficulties with activities of daily living, and impaired quality of life [1–6]. The prevalence of cerebellar ataxia is estimated to be 8.3 in 100,000 [7] and as high as 26 in 100,000 in children [8]. The prevalence of dominant hereditary cerebellar ataxia is 2.7 in 100,000, and the prevalence of recessive hereditary cerebellar ataxia is 3.3 in 100,000 [9]. Hereditary cerebellar ataxias can be due to triple repeat expansions or mutations of different genes in various types of spinocerebellar ataxias, Friedrich’s ataxia, ataxia-telangiectasia, dentatorubral-pallidoluysian atrophy, episodic ataxias, and ataxia with oculomotor apraxia [3]. Acquired cerebellar ataxia is more common than hereditary causes and is most often due to stroke, tumor, demyelination, or inflammation [3]. Ataxia can also manifest in the setting of neurodegeneration of multiple central nervous system pathways (e.g. multiple systems atrophy type C), paraneoplastic syndromes, and autoimmune disease. Presentations of cerebellar ataxia can be acute, episodic, subacute, or chronic, and can shorten life expectancy [10].

Patients with cerebellar ataxia complain of balance issues, incoordination, dysarthria, dysphagia, and peripheral weakness in addition to neuro-ophthalmic symptoms such as oscillopsia and difficulty with saccadic reading [2, 11–18]. Oscillopsia is an illusion of movement in the visual environment due to difficulty holding the eyes still, moving the eyes, and coordinating eye movement in general [4]. Difficulty reading can be due to abnormal accuracy of saccades (hypo- or hypermetria) and fixation instability (macrosaccadic oscillations or nystagmus), seen in many patients with cerebellar ataxia [1, 3, 4, 12, 19]. These saccadic and fixation abnormalities may be followed by corrective eye movements in order to appropriately target gaze positions and compensate for the retinal slip causing visual blur and reduced visual acuity [20–25].

To read and survey the visual surrounding, human beings make about 250,000 eye movements per day, rapidly jumping from target to target, and each time placing gaze on the fovea, the part of the retina with the highest resolution [21, 26–28]. Reflexive saccade and static fixation paradigms are traditionally used in infrared oculography studies to quantify saccade and fixation abnormalities in patients with cerebellar ataxia [25, 29–31]. However, these relatively simplified tests do not simulate the pattern of eye movement during functional tasks such as reading, which requires complex coordination of multiple parts of the brain to perform rapid, alternating saccades and fixations along each line of reading followed by line-changing saccades [21]. In order to perform such an ocular motor task well, the cerebellum optimizes saccadic and fixational behavior in sequence and integrates information from each saccade to plan the next one [21, 32–35]. This means patients with difficulty with eye movement control due to cerebellar ataxia may have particularly striking difficulties with reading, which is a reliable predictor for assessment of visual acuity, although this is not routinely measured in the clinical setting [31, 36].

The King-Devick test, a rapid number reading task that is read aloud, has been used to assess reading ability and ocular motor performance in patients with concussions [37, 38], multiple sclerosis [39, 40], Parkinson’s disease [39, 41], and Alzheimer’s disease [42–45]. However, this test has not yet been used to assess reading performance in patients with cerebellar ataxia. In this study, we evaluated reading performance using 3 different saccadic reading tasks in a heterogeneous group of patients with cerebellar ataxia and age-matched controls. Using infrared oculography, we quantified changes in ocular motor behavior by examining saccade and fixation characteristics that underlie the reading difficulties in patients with cerebellar ataxia.

## Materials and methods

### Participants

All experimental procedures were approved by the Stanford Institutional Review Board (IRB) and were in accordance with the Declaration of Helsinki and Health Insurance Probability and Accountability Act (HIPAA) regulations. Written informed consent was obtained from all participants after a test administrator explained the purpose of the study and how the tests are performed.

We performed a prospective, case-control study of 11 patients with cerebellar ataxia (mean age 54.0 ± 5.2 years, range 28–78 years, 54% women) (Table 1) and 11 age-matched controls (mean 50.4 ± 5.4 years, range 29–79 years, 73% women) recruited from the Stanford Neuro-Ophthalmology Clinic. Inclusion criteria were patients with inherited, genetically confirmed cerebellar ataxia or acquired cerebellar ataxia diagnosed by a neurologist or those with confirmed cerebellar lesions on brain imaging. Patients with acquired cerebellar mass all had their lesion surgically removed at least 6 months prior to recording. All participants had good visual acuity, no cognitive difficulties impacting reading, completed at least 8^th^ grade education, and had no history of developmental dyslexia or other reading difficulties. Control participants had no history of neurologic or ophthalmic symptoms or reading difficulty. Exclusion criteria were poor visual acuity, inability to read from a computer monitor, inability to hold head and posture well to perform the tasks, difficulty obtaining good recordings due to corrective lenses, visual field defect, other neurological or ophthalmic issues, or other causes of reading difficulty. All participants were permitted to wear their own corrective lenses or contacts during the study as long as they did not interfere with the quality of the recording.

**Table 1:** Demographic data for patients with cerebellar ataxia (n = 11).

Patient #	Diagnosis	Gender	Age	Side of involvement
1	Spinocerebellar Ataxia Type 6	M	74	B
2	Multiple Systems Atrophy	M	54	B
3	Hereditary Ataxia	M	78	B
4	Cerebellar Hemangioblastoma (Von Hippel-Lindau)	F	31	B
5	Pilocystic Astrocytoma	F	28	R
6	Cerebellar Astrocytoma	M	34	L
7	Cerebellar Stroke	F	48	R
8	Paraneoplastic Ataxia	F	68	B
9	Autoimmune Cerebellar Ataxia	M	68	B
10	Spinocerebellar Ataxia 42	F	57	B
11	Idiopathic Cerebellar Ataxia	F	54	B

Abbreviations: M: male, F: female, B: bilateral, L: left, R: right

Patients with cerebellar ataxia had mean visual acuity of 20/20 in the better seeing eye (LogMAR range: 0–0.18), exhibited no relative afferent pupillary defect, and had intraocular pressures and optic nerve measurements within normal limits. Average disease duration was 8.2 ± 2.7 years (range: 0.3–26 years). The patients reported symptoms of oscillopsia (5, 45%), balance instability (5, 45%), binocular diplopia at distance (4, 36%) or at near (3, 27%), dizziness (3, 27%), headache (3, 27%), neck discomfort (2, 18%), and dysarthria (4, 36%). On ocular motor examination, patients with cerebellar ataxia exhibited hypermetric or hypometric saccades (9, 82%), saccadic pursuit (5, 45%), gaze evoked nystagmus (5, 45%), saccadic oscillations or square wave jerks (4, 36%). Patients had ocular misalignment at primary gaze at distance (7, 64%; all small misalignments except for one and not necessarily symptomatic) or at near (2, 18%; one patient did not have alignment measured at near), which were assessed using an alternating cover uncover test to measure prism diopter of misalignment. None of the patients had alternating skew deviation. One patient (#3) had 2 Hz small continuous square wave jerks to the left, another (#8) had mild fixation instability with episodic brief bursts of saccadic intrusions, and another (#11) had a small 1–2 Hz 3-dimensional nystagmus most prominently down-beating in primary gaze.

### Saccadic reading tasks

**Fig 1:**
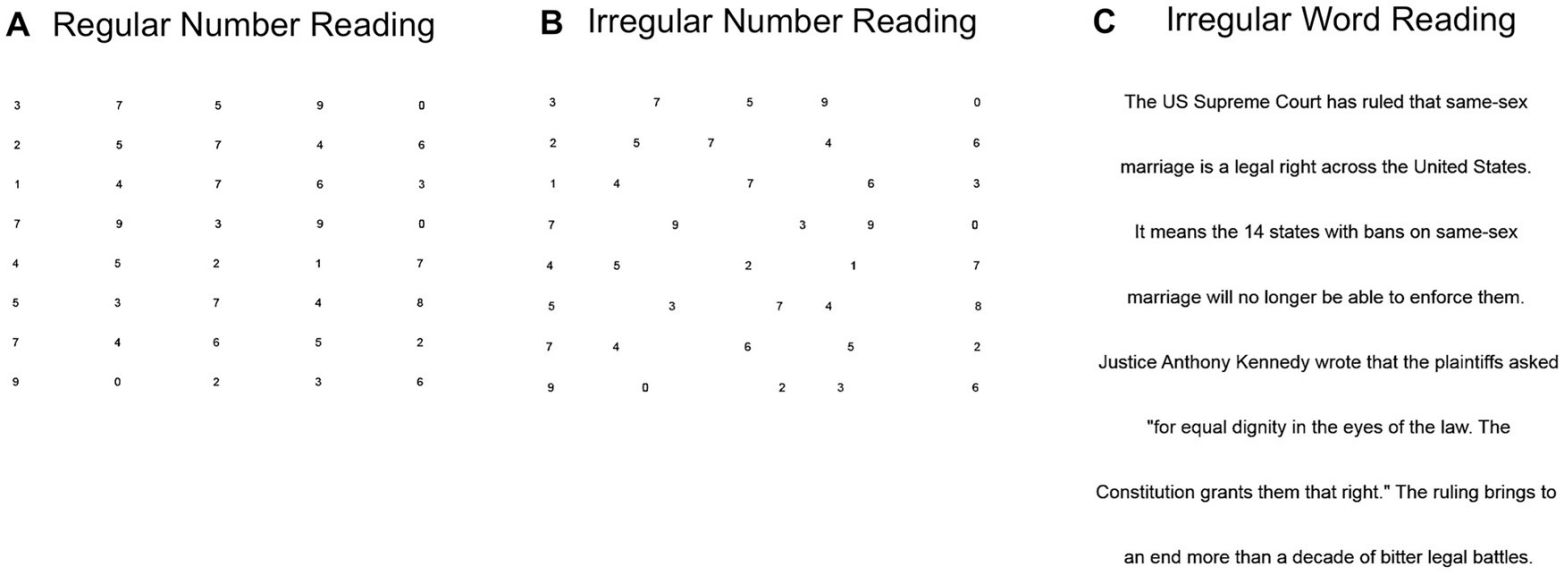
Examples of the 3 saccadic reading tasks. (A) 40 regularly spaced single-digit numbers read aloud (2^nd^ page) (B) 40 irregularly spaced single-digit numbers read aloud (2^nd^ page, King-Devick test) (C) 74 irregularly spaced words in sentences with semantic context read silently (natural reading, 3^rd^ page).

We used 3 different left-to-right saccadic reading tasks each consisting of 3 pages per task (total 9 pages) performed in the same order once: 1) 120 regularly spaced single-digit numbers read aloud (3 pages of 40 single digit numbers/page, 5 numbers or 60 characters + spaces/line in Arial size 18, 8 lines/page), 2) 120 irregularly spaced single-digit numbers read aloud (same dimensions and numbers as task #1 except irregularly spaced in the same way as the King-Devick test) [38], and 3) 188 irregularly spaced words read silently (natural reading, 3 pages of words, 24–55 characters + spaces/line in Arial size 36, 8 lines/page, Fig 1). The font size of the two number reading tasks was smaller than the word reading task in order to match the King-Devick test booklet when shown on the computer monitor. All font sizes were 18 or greater to ensure font size would not impact reading speed. The two number reading tasks were performed aloud to ensure participants read all number correctly—the original King-Devick test is also performed aloud [38]. The word reading task was completed silently to simulate natural reading. An initial demonstration page on paper was used to show subjects how the test is performed, from left-to-right and top-to-bottom, and opportunities to practice were offered. Participants were asked to read as fast as possible without making errors, and they were aware that this was a timed test. Prior to initiating the test, a demonstration page was presented to the participants on the computer as practice if needed.

### Eye movement recording

We performed two-dimensional 500-Hz binocular video infrared oculography (RED500, SensoMotoric Instruments, Germany). The RED500 is a contact-free system that automatically compensates for small head movements and can be used without a chin rest in cooperative subjects. The gaze point in the stimulus was determined by tracking the corneal light reflex relative to pupil center.

Subjects sat on an armchair approximately 70 cm in front of a computer monitor (horizontal and vertical dimensions: 55.9 cm by 33.0 cm, 22 inches by 13 inches, 1680 px by 1050 px). Each subject performed a 9-point calibration task binocularly including cardinal points of regard in order to track gaze during reading. Multiple validation steps were performed between all tasks using fixation points to ensure high quality and accuracy of recording. Deviations in the x and y dimensions were calculated by measuring gaze points with the known calibration target location. We did not examine individual eye movements because none of these patients had disconjugate eye movements. Subjects were instructed to sit in a consistently upright position with eyes open and to minimize head movement throughout the test. When necessary, participants were repositioned and their heads may be stabilized by the test administrator to minimize head movement during recording. A chin rest was not used because some reading tasks were performed aloud. Eye movement recording procedures were explained to all participants prior to the test. During the test, an investigator vigilantly tracked errors and troubleshot any technical issues. Progressive lenses and glasses with prisms can interfere with eye tracking, so the recording was stopped or not used if the quality of recording was poor. Only a few subjects wore their own glasses during testing, and, although this was not intentional, none of the ataxia patients wore glasses during recording. If necessary, the recording was re-started to ensure the best quality and to minimize errors. Infrared oculography was used to ensure subjects performed all the tasks well and did not skip lines. Infrared oculography recordings were optimized for high tracking ratios and reading accuracy.

### Data analysis

All ataxia patients (n = 11) and control participants (n = 11) completed the regularly and irregularly spaced number reading task. One ataxia patient was excluded from analysis of the regularly spaced number reading task due to lost tracking in one of the pages (n = 10). All 11 control participants completed the regularly spaced number reading task with accurate tracking. Five ataxia patients and 9 controls completed the word reading task and had high quality recordings.

Each recording was reviewed to ensure accurate calibration and consistent eye and head position. We reviewed individual scan path videos for all tasks and rejected any recordings with poor calibration, loss of more than half a line of reading data, or artifacts related to head or body movement. Gaze paths were first reviewed for quality assurance. Gaze recordings with tracking ratios under 90% were re-evaluated by examining the scan path videos again. Any videos with a tracking ratio less than 90% and increased noise and artifacts were excluded. The tracking ratio during the entire experiment was on average 94.8 ± 1.5% in controls and 96.4 ± 0.7% in the ataxia patients. In controls, the mean deviation x was 0.7 ± 0.2°, and deviation y was 0.5 ± 0.1°, while in ataxia patients, mean deviation x was 0.9 ± 0.2°, and deviation y was 0.8 ± 0.1°.

We measured total reading time for each of the three reading tasks by adding reading time for the three pages of regularly spaced numbers, three pages of irregularly spaced numbers, and three pages of irregularly spaced words. We calculated overall reading speed per second for the three tasks using the formula: reading speed = 120/total reading time for each number reading task and reading speed = 188/total reading time for the word reading task. Total reading time and reading speeds were compared between ataxia patients and controls for the three tasks. For each task, differences in mean reading speed between controls and ataxia patients and the relative change (%) in reading speed or reading time was calculated: [(mean reading speed in controls—mean reading speed of ataxia)/mean reading speed in ataxia] or [(total reading time in ataxia—total reading time in controls)/total reading time in controls].

Data from the 120 regularly spaced single-digit number reading task was further analyzed using commercially available software (BeGaze, SensoMotoric Instruments, Germany) and custom written code in Python. A saccade was defined as a change in gaze between two points with a minimum peak velocity threshold of 40°/s and minimum duration of 22 ms. A fixation was defined as a gaze with velocity below 40°/s (not a saccade) with minimum duration of 50 ms. We measured the total time spent making saccades and fixations in seconds and calculated the percentage or proportion of time making saccades and fixations (i.e. total time in saccades / total reading time). Total saccade and fixation count and scan path lengths were also measured, and mean values calculated from individual measurements were compared between ataxia patients and controls. For saccade analysis, we quantified the mean saccade amplitude (°), velocity (°/s), and duration (ms) for each participant (total saccade amplitude or velocity or duration / total saccade count) and compared values between ataxia patients and controls. For the main sequence relationships, we pooled all saccades made by control subjects and plotted all saccade amplitude vs. duration and amplitude vs. peak velocity. The same was done for saccades made by ataxia patients. The relative frequency distribution was plotted for saccade amplitudes (bin width: 0.5°), peak velocities (bin width: 10°/sec), and durations (bin width: 5ms) in patients and controls. For fixation analysis, we calculated the mean fixation duration (ms), dispersion (px), and x- and y- dispersion (px). Mean fixation duration and dispersion were calculated for each participant (total fixation duration or dispersion / total fixation count) and compared between groups. Individual duration and x- and y- dispersion values were pooled for each group and compared between groups. Dispersions greater than 100 pixels were excluded from analysis. Individual fixations were pooled into two groups to compare mean duration and x- and y- dispersions between controls and ataxia patients. Relative frequency distribution power analyses for fixation duration (bin width: 50 ms), and x- (bin width: 5 px) and y- dispersion (bin width: 5 px) were plotted.

To evaluate scan path pattern during saccadic reading, we exported and reviewed all scan path videos, which also helped ensure high quality of the calibration and recording. To assess gaze pattern, we plotted the horizontal gaze position over time during saccadic reading to visualize the staircase patterns during saccadic reading, with the vertical lines indicating rightward, small progressive saccades and horizontal lines corresponding to fixation duration. Spatial deviations were calculated using a custom Python script, which sorted all fixations into one of 5 regularly spaced columns or regions of interest and calculated the x and y deviations of the fixations from the number target positions using the formula: ([Fixation position (x, y)—Target position (x, y)]). Mean spatial deviation was calculated for each participant and compared between groups.

### Statistical analysis

Due to the small sample size, we performed nonparametric statistical analysis using the Mann-Whitney U test. For the three reading tasks, we compared reading speed between controls and ataxia. A linear regression model was used to correlate reading speeds between the three reading tasks for all participants together. A repeated measures two-way ANOVA was completed by fitting a linear mixed effects model to analyze reading speed. For the regularly spaced number reading task, total time in saccades and fixations, percentage of time in saccades and fixations, saccade and fixation count, and total scan path length in pixels were measured for each participant, and the mean value for each group was calculated by averaging each participant’s measurements. A linear regression model was used to describe the relationship between reading time and saccade count, fixation count, and scan path length for all participants and to correlate relationship between saccade amplitude and duration for each group. A one phase exponential equation estimated the relationship of saccade amplitude and peak velocity (V_peak_ = V_max_ [1−e^(−amplitude/C)^], V_max_ = asymptotic peak velocity, C = angle constant). All statistical analyses were performed with Prism software (GraphPad), and statistical significance was set at p < 0.05.

## Results

### Ataxia patients read significantly slower than age-matched controls during regularly and irregularly spaced number reading tasks

**Fig 2:**
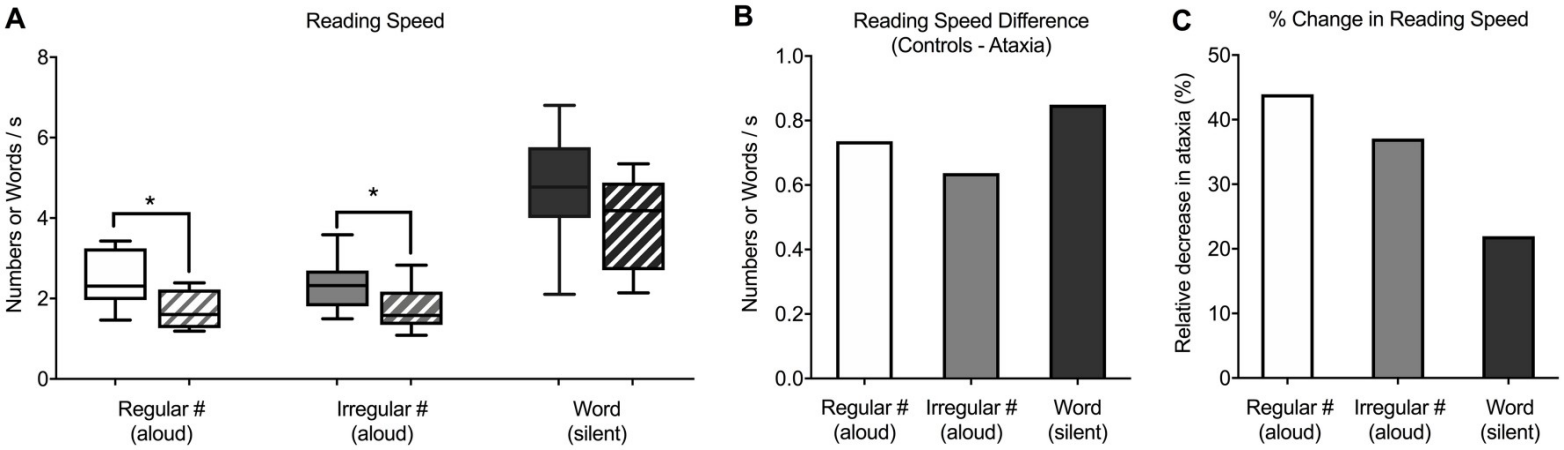
Reading speed in cerebellar ataxia patients and age-matched controls during the three saccadic reading tasks. (A) Box-whisker plots of reading speed (numbers/s or words/s) in age-matched controls (solid) and patients with cerebellar ataxia (stripes). There was significantly slower reading speed in ataxia patients during regularly spaced (p = 0.02) and irregularly spaced number reading (p = 0.02). (B) Bar graph of difference in reading speed (controls—ataxia). (C) Bar graph of relative difference (%) in reading speed in ataxia patients compared with controls. White = regularly spaced number reading aloud, grey = irregularly spaced number reading aloud (King-Devick test), black = irregularly spaced word reading silently.

We recruited 11 patients with different causes of cerebellar ataxia and 11 age-matched controls to perform 3 saccadic reading tasks (see Materials and methods). Patients with cerebellar ataxia read significantly slower than age-matched controls during the regularly spaced (ctrl: 2.4 ± 0.2 numbers/s or reading time of 53.6 ± 4.7 s, ataxia: 1.7 ± 0.1 numbers/s or reading time of 76.1 ± 5.6 s; 44% slower; p = 0.02, U = 24) and the irregularly spaced single-digit number reading tasks (ctrl: 2.4 ± 0.2 numbers/s or reading time of 54.1 ± 4.2 s, ataxia: 1.7 ± 0.2 numbers/s or reading time of 75.3 ± 6.5 s; 37% slower; p = 0.02, U = 22) (Fig 2A). Reading speed was slower in ataxia patients during silent word reading (Fig 2A and 2B), but this difference was not significant (ctrl: 4.7 ± 0.5 words/s or reading time of 44.2 ± 6.1 s, ataxia: 3.9 ± 0.5 words/s or reading time of 53.7 ± 9.3 s; 22% slower; p > 0.05, U = 15). When fitting with a linear mixed effects model to analyze using repeated measure ANOVA, we found that ataxia patients had reading speeds significantly slower than those of controls (-0.72 ± -0.31; p = 0.03). Reading speeds on the irregularly spaced number reading task were similar to those on the regularly spaced number reading task (0.0 ± 0.09), and there was no indication of a diagnosis-task interaction (p > 0.05).

Of the three reading tasks, the performance on the regularly spaced number reading task was the most different (44% slower compared with 37% in irregularly spaced number reading and 22% in word reading) between ataxia and control groups (Fig 2C). Reading speeds during regularly spaced number reading was significantly correlated with that of the irregularly spaced number reading (R^2^ = 0.75, p < 0.0001) and silent word reading (R^2^ = 0.80, p < 0.0001).

### Slower reading during regularly spaced number reading due to more time making eye movements and higher number of saccades

**Fig 3:**
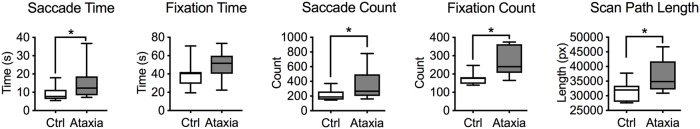
Ataxia patients made more saccades and fixations and had greater scan path lengths while reading 120 regularly spaced single-digit numbers aloud. Box-whisker plots showing that ataxia patients (grey) had increased saccade time (p = 0.02), fixation time (p > 0.05), saccade count (p = 0.01), fixation count (p = 0.004), and scan path length (p = 0.01) compared with control subjects (white).

Since the regularly spaced number reading task showed the biggest difference between the ataxia and the control groups, we further analyzed the infrared oculography data to determine why ataxia patients read slower while performing this task. Compared with controls, ataxia patients spent 69% more time making saccades (ctrl: 9.1 ± 1.2 s, ataxia: 15.4 ± 2.9 s; p = 0.02, U = 26), and 23% more time making fixations (ctrl: 40.5 ± 4.8 s, ataxia: 50.0 ± 4.2 s; p > 0.05, U = 37, Fig 3). This meant that ataxia patients spent approximately 6% more of their total reading time making eye movements (ctrl: 17.6 ± 2.3%, ataxia: 23.5 ± 4.0%; p > 0.05, U = 41) and 7% more of their total time in fixation compared with controls (ctrl: 74.6 ± 4.0%, ataxia: 67.3 ± 5.1%; p > 0.05, U = 47). While reading 120 numbers, ataxia patients made 78% more saccades (ctrl: 213.9 ± 24.1, ataxia: 381.7 ± 67.0; p = 0.01, U = 24), 49% more fixations (ctrl: 174.4 ± 9.2, ataxia: 259.6 ± 23.4; p = 0.004, U = 18), and 17% longer total scan path lengths (ctrl: 31,601 ± 1031 pixels, ataxia: 36,919 ± 1548 pixels; p = 0.01, U = 22, Fig 3). Reading time was significantly correlated with saccade count (R^2^ = 0.37, p = 0.002), fixation count (R^2^ = 0.73, p < 0.0001), and scan path length (R^2^ = 0.58, p < 0.0001).

### Ataxia patients made smaller and slower saccades during regularly spaced number reading task

**Fig 4:**
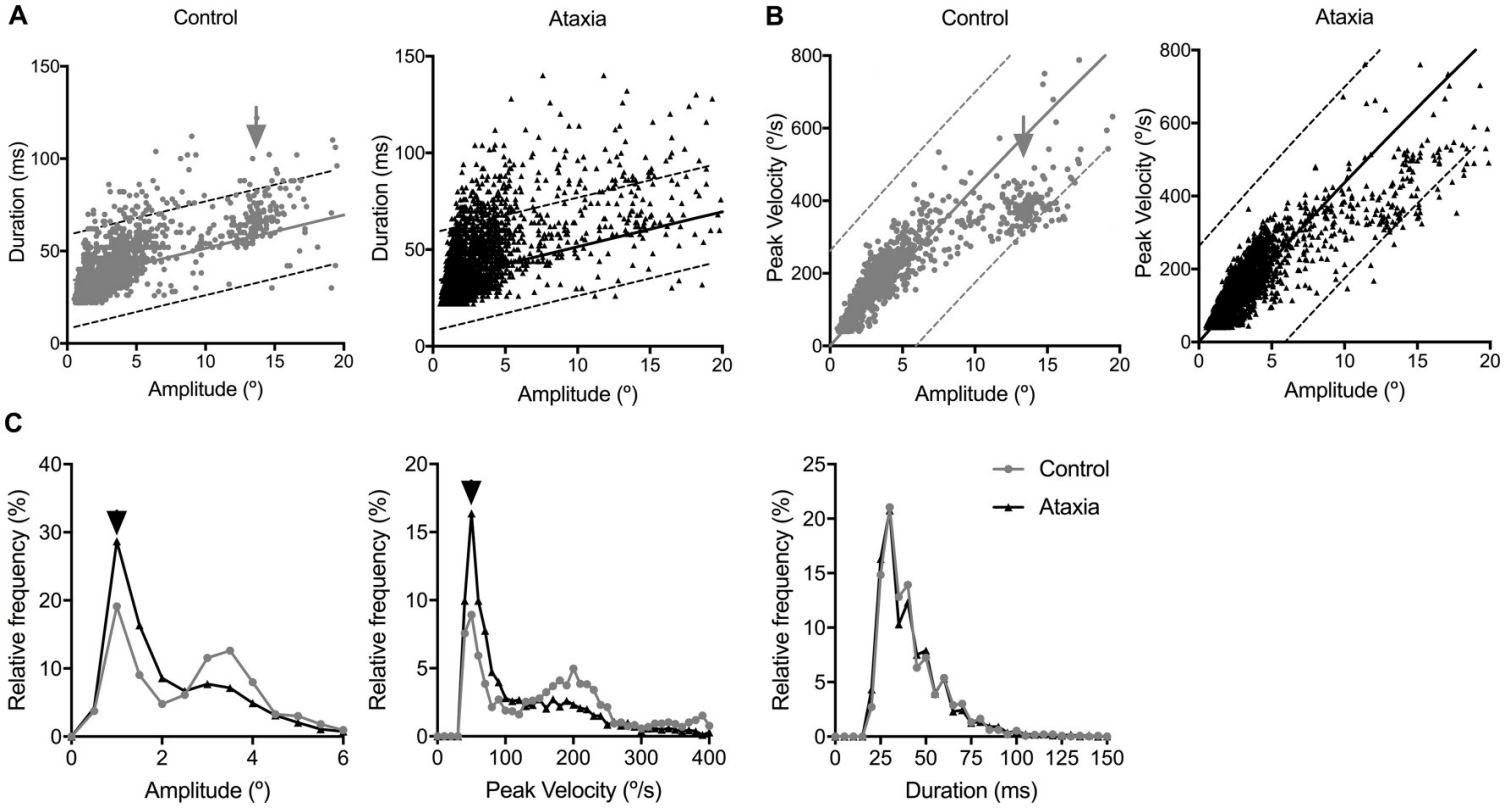
Saccades were irregular and abnormal in ataxia patients compared with controls while reading 120 regularly spaced single-digit numbers aloud. (A) Main sequence relationship scatter plots of saccade amplitude (°) vs. duration (ms) and (B) saccade amplitude (°) vs. peak velocity (°/s) for controls (grey) and ataxia patients (black). In controls, there was a clear cluster of larger amplitude line-changing saccades (black arrows) that was less prominent in ataxia patients. A linear regression model (saccade amplitude vs. duration) and one phase exponential equation (saccade amplitude vs. peak velocity) was used to fit data from saccades by controls with 5% and 95% prediction bounds dotted. (C) Relative frequency power analysis of saccade amplitude (°), peak velocity (°/s), and duration (ms) for controls and ataxia patients showed that compared with that of controls, the peak relative frequency of saccade amplitude and peak velocity was smaller and slower, respectively, in ataxia patients (black arrowheads).

While patients with cerebellar ataxia made more saccades during regularly spaced number reading, their average saccade amplitudes were significantly smaller by 29% (ctrl: 5.0 ± 0.4°, ataxia: 3.6 ± 0.3°; p = 0.01, U = 22) and slower by 25% (ctrl: 103.3 ± 8.1°/s, ataxia: 77.6 ± 6.6°/s; p = 0.05, U = 30). There was no significant difference in saccade duration between controls and patients with ataxia (ctrl: 42.1 ± 1.0 ms, ataxia: 40.9 ± 2.0 ms; p > 0.05, U = 53). The same findings were seen on main sequence relationships of saccade amplitude and duration (Fig 4A), saccade amplitude and peak velocity (Fig 4B), and relative frequency power analyses of saccade amplitude, peak velocity, and duration (Fig 4C). In addition, main sequence relationships of saccades in control subjects showed an obvious clustering of small saccades that are distinct from the larger amplitude, faster velocity line-changing saccades (Fig 4A and 4B). This clustering of line-changing saccades was not as obvious in patients with cerebellar ataxia, consistent with abnormal line-changing saccades. This nice segregation of small and large amplitude saccades in control subjects and less distinct clustering of large, line-changing saccades in ataxia patients were also seen in plots of individual subjects (not shown). In all participants, saccade amplitude significantly correlated with duration (ctrl: R^2^ = 0.48, p < 0.0001; ataxia: R^2^ = 0.41; p < 0.0001) and peak velocity (ctrl: R^2^ = 0.78; p < 0.0001; ataxia: R^2^ = 0.79; p < 0.0001).

### Ataxia patients made more fixations with shorter duration and greater dispersion during regularly spaced number reading

**Fig 5:**
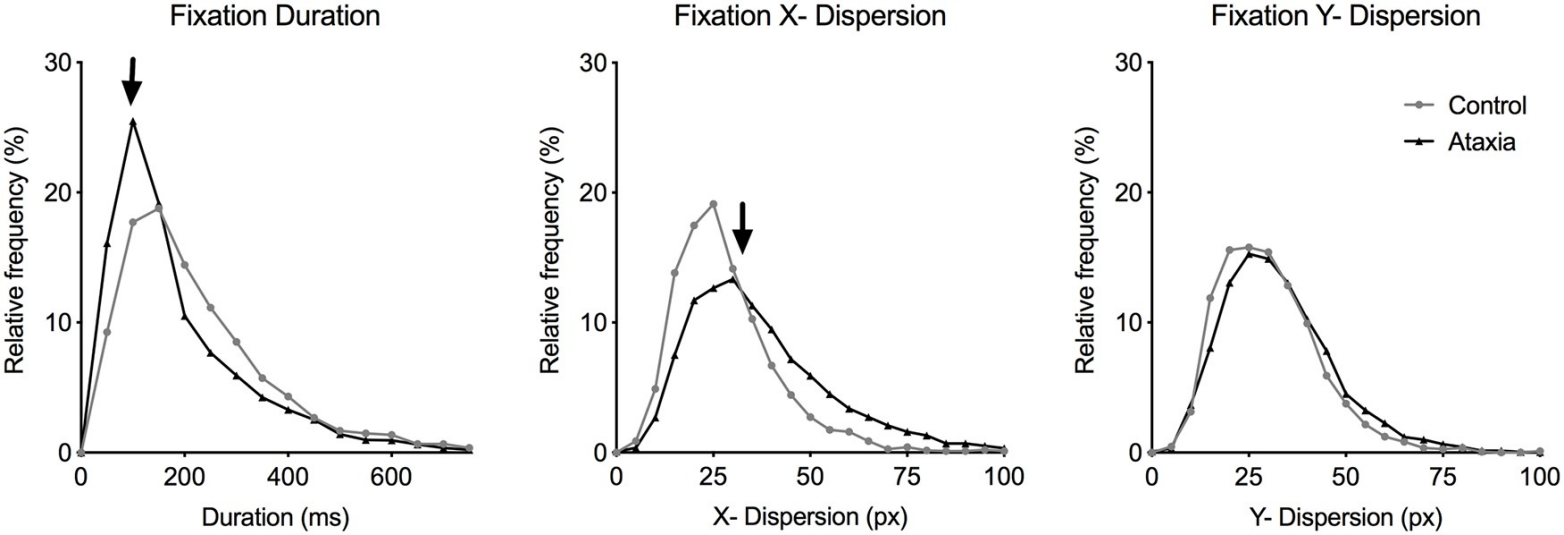
Ataxia patients made more fixations with shorter durations and greater dispersion while reading 120 regularly spaced single-digit numbers aloud. Relative frequency power analysis of fixation duration (ms) and x- and y-dispersion (px) for controls (grey line) and ataxia patients (black line). Compared with controls, ataxia patients had a relatively greater peak at shorter fixation duration (left, arrow), greater x-dispersion (middle, arrow), and no difference in y-dispersion (right) during regularly spaced number reading.

During regularly spaced number reading, ataxia patients made significantly more fixations by 49% (ctrl: 174.4 ± 9.2, ataxia: 259.6 ± 23.4; p = 0.004, U = 18, Fig 3) but did not spend more time in fixations because the fixations were on average shorter in duration by 30 ms compared with controls when average fixation durations were calculated for individual subjects (ctrl: 230.2 ± 23.2 ms, ataxia: 200.9 ± 18.5 ms; p > 0.05, U = 52). The average fixation dispersions for individual subjects were only a few pixels greater in the ataxia group compared with controls (ctrl: 71.7 ± 5.4 px, ataxia: 76.0 ± 5.5 px; p > 0.05, U = 50), consistent with overrepresentation of some subjects with greater fixation dispersion in the pooled analysis. Pooled fixation measurements showed that fixations in ataxia patients were shorter in duration (ctrl: 232.5 ± 3.8 ms, ataxia: 192.5 ± 2.7 ms; p < 0.0001, U = 2305501), and had greater x- (ctrl: 28.3 ± 0.3 px, ataxia: 36.8 ± 0.3 px; p < 0.0001, U = 1902557) and y- dispersion (ctrl: 30.1 ± 0.3 px, ataxia: 32.6 ± 0.3 px; p < 0.0001, U = 2481490). Similar results were seen using relative frequency analysis of fixation duration and x- and y- dispersion (Fig 5). Total reading time significantly correlated with total fixation count (R^2^ = 0.73, p < 0.0001).

### Irregular scan paths and staircase gaze pattern during regularly spaced single-digit number reading in ataxia

**Fig 6:**
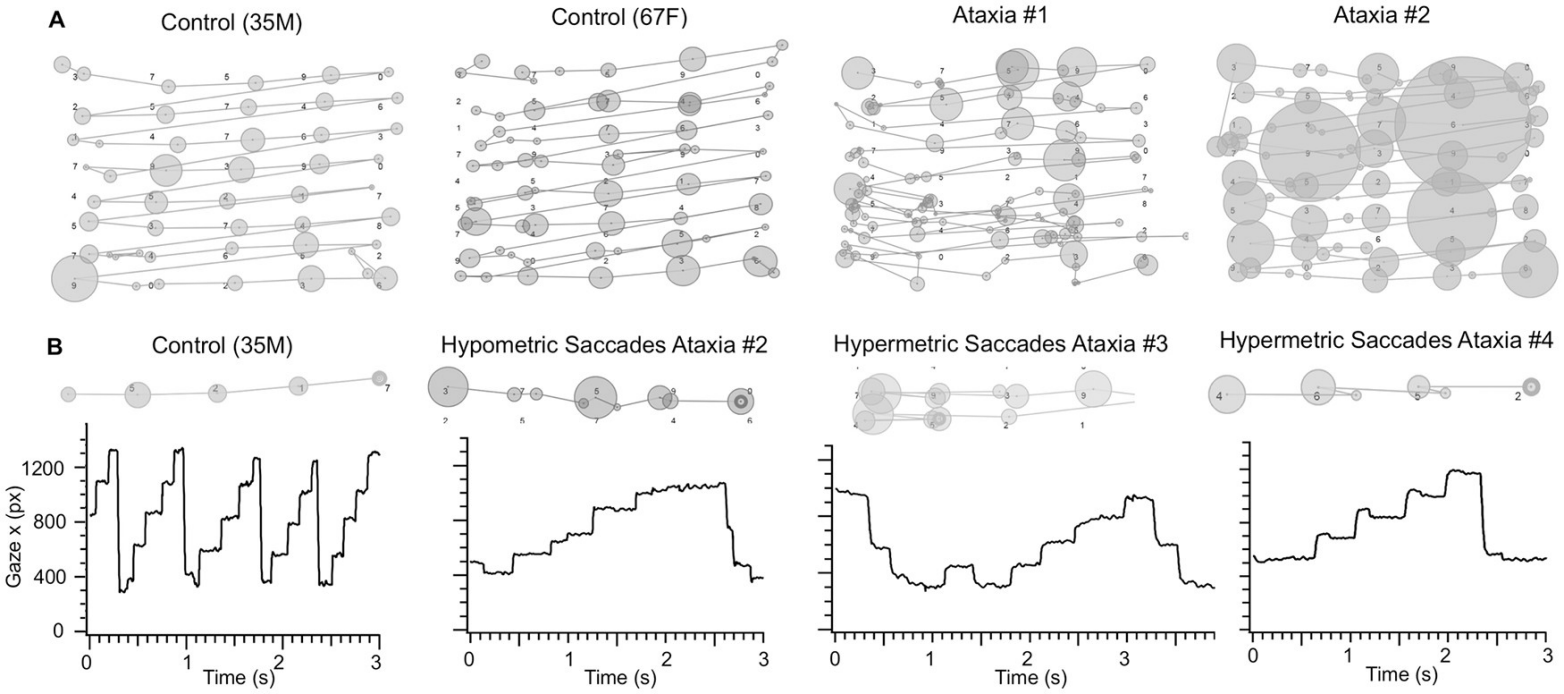
Irregular scan paths and staircase gaze patterns in ataxia patients during regularly spaced single-digit number reading. (A) Scan paths during regularly spaced number reading (2^nd^ page) of a 35-year-old male control, 67-year-old female control, ataxia patient #1 (spinocerebellar ataxia type 6), and ataxia patient #2 (multiple system atrophy). Circles denote fixations, size of the circles corresponds with duration, and lines indicate saccade paths between fixations. (B) Line graph of horizontal gaze position during regularly spaced number reading (2^nd^ page) of a 35-year-old male control (left) and ataxia patients #2–4 (see Table 1). Vertical lines indicate saccades to the right, and horizontal lines indicate fixation. Corresponding segments of scan paths are shown above. Scale: y-axis long ticks = 400 px, x-axis long ticks = 0.5 sec.

Consistent with slower reading and changes in saccade and fixation parameters, ataxia patients also exhibited more irregular scan paths and staircase pattern of horizontal (x) gaze positions while reading 120 regularly spaced single-digit numbers aloud. While the scan paths of control subjects were organized with small circles (corresponded with short fixation durations) alternating between clear lines (saccades) that connect the circles, the scan paths of ataxia patients had circles of varying size (corresponded with irregularity of fixation durations) and disorganized lines (abnormal saccades, Fig 6A). Line graphs of x gaze position also revealed that control subjects made clear progressive saccade steps from left to right in a regular staircase-like pattern, while ataxia patients made a variety of irregular staircase patterns (Fig 6B). Faster reading in controls corresponded with presence of multiple staircase patterns in the same amount of time, and slower reading in ataxia patients corresponded with broader staircase steps horizontally (slower reading: Ataxia #2, #3, #4, see Table 1), and irregular, short steps vertically (irregular, smaller progressive saccades: #2). While line-changing saccades are typically large and involving a single step in controls, line-changing saccades may involve smaller saccades and multiple steps in ataxia patients (hypometric line-changing saccades: #2, #3). Ataxia patients sometimes had extra steps at the start of the line (fixation instability: #3) or overshooting steps (hypermetria in progressive saccades: #4), consistent with poor eye movement control (Fig 6B). Poor eye movement control also manifested as increased spatial deviation of fixations from target numbers by approximately 8 pixels greater in ataxia patients compared with controls (x-axis deviation: ctrl: 23.5 ± 1.2 px ataxia: 31.1 ± 4.4 px; U = 25, p > 0.05; y-axis deviation: ctrl: 40.1 ± 5.5 px, ataxia: 48.2 ± 9.1 px; U = 41, p > 0.05).

### Irregular scan paths and staircase gaze pattern during silent word reading in ataxia

**Fig 7:**
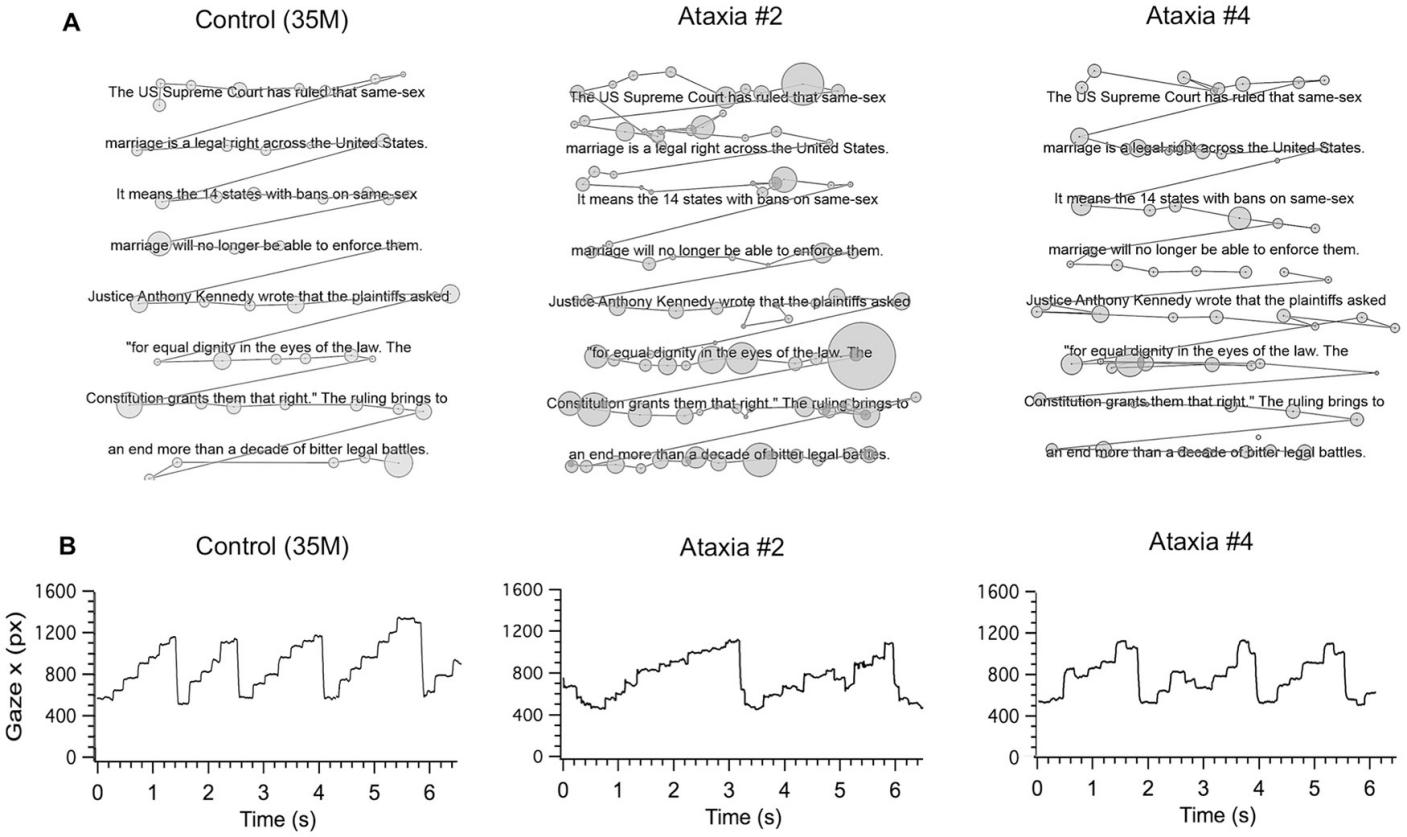
Irregular scan paths and staircase gaze patterns in ataxia patients during silent word reading. (A) Scan paths during word reading (3^rd^ page) of a 35-year-old male control, ataxia patient #2 (multiple systems atrophy) and ataxia patient #4 (cerebellar hemangioblastoma). Circles denote fixations, the size of the circles corresponds with duration, and lines indicate saccades between fixations. (B) Line graphs of horizontal gaze position during silent word reading revealed similarly abnormal hypometric or hypermetric saccades in ataxia patients during word reading compared with number reading (Fig 6).

To determine whether irregularity of scan paths and staircase gaze patterns also exists in natural silent word reading, we analyzed data from 5 ataxia patients (# 2, 4, 5, 7, 8) and 9 controls during a silent word reading task, which consisted of 188 irregularly spaced words with semantic context (Fig 1C). There was a qualitatively similar pattern of irregular scan paths (irregularity of fixation durations as denoted by the different sizes of the circles and irregular length of the saccades as denoted by different lengths of lines) in ataxia patients in the number reading task (Fig 6A, ataxia patient #2) vs. the word-reading task (Fig 7A, ataxia patient #2). This was strikingly different from the regular scan path pattern of the controls (Figs 6A and 7A, 35-year-old male control). Ataxia patients also made qualitatively similar abnormal staircase pattern of x gaze position during number and word reading (Figs 6B and 7B, ataxia patient #2 and #4). For example, ataxia subject #2 made hypometric, progressive saccades during both number and word reading, while ataxia patient #4 consistently made hypermetric progressive saccades during both number and word reading.

## Discussion

The hallmark of eye movement abnormality in cerebellar ataxia is abnormal saccades and fixation instability, which impact the over 60,000 eye movements made during an 8-hour workday or the 250,000 eye movements during a 24-hour period [28]. Poor eye movement control can cause eye and mental fatigue that worsens later in the day (unpublished observation) and leads to impaired activities of daily living such as reading, resulting in worse quality of life [6, 29, 46–50]. Therefore, it is not surprising that patients with cerebellar ataxia have difficulty with reading [31, 36]. Because improved reading has been reported as the number one goal or complaint in patients with vision difficulties [51–53], assessment of reading should be more routinely performed in the clinical setting. Simple number reading tests such as regularly (this study, [54]) or irregularly spaced (King-Devick test, [37, 39–41]) number reading tests can rapidly and accurately quantify reading speed in patients with different neurological conditions in the clinic. A regularly spaced task involving small saccades like the one used in this study may be particularly suitable for measuring patients with cerebellar ataxia, since patients with cerebellar ataxia tend to have difficulty making precise, small saccades as well as performing repetitive tasks.

Our infrared oculography study revealed that patients with cerebellar ataxia have significant difficulties making the ocular motor patterns necessary during reading, which is the reason for slower reading in cerebellar ataxia. While infrared oculography remains the gold standard for such assessment, this type of measurement remains difficult to obtain for most clinicians. In the clinical setting, measurement of reading speed using the 120 single-digit number reading tests, which can be completed in less than 2 minutes by most patients, is a useful quantification of ocular motor difficulties in patients with cerebellar ataxia.

In our study, we examined ocular motor behavior in patients with cerebellar ataxia and age-matched controls during three reading tasks: 1) regularly and 2) irregularly spaced number reading aloud and 3) irregularly spaced word reading silently. Compared with controls, patients with cerebellar ataxia had the greatest decrease in reading speed by 44% while performing the easiest, simplest task—the 120 regularly spaced numbers. This is because reading regularly spaced single-digit numbers is easier and faster than irregularly spaced numbers in controls [54]. In contrast, patients with cerebellar ataxia have great difficulty making precise, *regular* patterns of *small* eye movement, and our data provide support for the utility of this simple saccadic reading task as a way to differentiate the ocular motor abilities in those with cerebellar ataxia from that of controls, to identify difficulties in reading performance in patients with cerebellar ataxia, and to monitor ataxia patients over time in the clinical setting.

Infrared oculography during regularly spaced single-digit number reading showed that slower reading in ataxia patients was due to abnormal saccade and fixation behavior, which are well reported with eye movement recording studies of reflexive eye movement and fixation in cerebellar ataxia [12, 29, 31, 55, 56]. These abnormalities lead to more frequent and abnormal saccades and fixations during reading [31, 34, 36] as well as other visual tasks [57]. In our study, ataxia patients globally made more saccades with smaller amplitudes and slower velocities and more fixations with shorter duration and greater dispersion compared with controls. However, these global fixation and saccade measurements do not tell the whole story because they are an average of a mixture of small progressive saccades, dysmetric saccades, and line-changing saccades. Closer examination of the individual scan paths and staircase patterns of horizontal and vertical gaze positions helps clarify the nuances of the different ocular motor abnormalities in different patients. Patients may have hypo- or hypermetric saccades; different types and severity of fixation instability; variable amount of corrective saccades after a small, progressive saccade or a large line-changing saccade; and abnormal line-changing saccades [1, 20, 23]. Abnormal staircase gaze pattern during reading is reported in cerebellar ataxia [36], Parkinson’s disease [41], and Alzheimer’s disease [36, 41, 43], and while shallow, longer, or irregularity of the steps in the staircase pattern is reported in all three conditions, patients with cerebellar ataxia have even more striking hypermetria and fixation instability [31, 36, 41, 57]. While reading regularly spaced numbers, patients with cerebellar ataxia exhibited increased number of saccades, fixations, and spatial deviation while reading 120 regularly spaced numbers, suggestive of saccadic dysmetria due to inadequate gaze control and errors in trajectory, consistent with that reported in previous studies [1, 2, 23, 29, 47, 56–59]. Fixation instability from square wave jerks, saccadic oscillations, ocular flutter, nystagmus, and microsaccades may also contribute to slow reading by increasing the amount of time spent making eye movements and the number of small saccades and brief fixations [12, 26, 57, 60–63].

Previous infrared oculography studies use relatively simple tasks such as reflexive saccades or fixation on a single point, which are excellent ways to assess the ability to generate these eye movements but do not adequately simulate the rapid and innumerable series of saccades and fixations that are part of our daily experience. Conversely, studies of saccadic word reading use complex paradigms that are more relevant to specific types of eye movement behavior and are unfortunately, influenced by semantic, syntactical, and phonological cues and higher order visual and cognitive abilities. There is currently no standardized assessment of reading performance in clinical practice, and we propose that the regularly spaced single-digit number reading task is a simple way to rapidly quantify ocular motor abilities needed for reading in patients with cerebellar ataxia. The single-digit saccadic reading task is more functionally relevant (to reading) than assessments of reflexive saccades or pursuit, although this test is not necessarily more able to detect a specific alteration of cerebellar function than measurements of reflexive saccade, vestibular ocular reflex, or smooth pursuit. Our results confirm the utility of a simple reading test to identify ocular motor impairment in those with cerebellar ataxia. This test can be done in less than one minute in controls and in less than 2 minutes in those with cerebellar ataxia. Reading aloud provided immediate feedback that participants performed the test correctly, without necessitating infrared oculography or a reading comprehension test. The addition of video infrared oculography is a useful, noninvasive way to better understand and quantify the efficacies and irregularities of small progressive saccades and large line-changing saccades as well as fixation behavior during reading. Visualization of the pattern of ocular motor abnormalities during reading can help direct pharmacologic therapies and determine most helpful reading rehabilitation strategies. With a larger study to assess the utility of single-digit number reading test and to compare with other assessments of cerebellar ataxia, we believe that a simple, regularly spaced single-digit number reading test may be a useful biomarker for patients with cerebellar ataxia in the clinical setting.

With silent word reading, ataxia patients read slower than controls, although this was not statistically significant in our small study, unlike the number reading tests and a previous study of 24 cerebellar ataxia patients by Alexandre *et al*., which showed that those with ataxia read significantly slower than controls while reading words silently [31]. This discrepancy is likely because of greater variability in the performance of the word reading task, small sample size, and potential confounding variables such as age, cognitive function, education, and other factors that affect word reading [31, 36]. A larger study in cerebellar ataxia patients will be important to better understand the difference between number and word reading.

Another possible explanation for the difference in the number and word reading tests is the contribution of speech and dysarthria, given the number reading tests were performed aloud while the word reading test was completed silently. Because reading aloud is the easiest way to verify that the subject performed the task in the clinical setting, these number reading tests are traditionally performed aloud. Although we did not specifically study the impact of dysarthria by comparing number reading speed aloud versus silently, we believe that dysarthria did not contribute significantly to the differences in the tasks read aloud because all patients had easily understood speech, had no difficulty reading single digit numbers, and we did not require perfect enunciation for the tests that are read aloud.

Because we are studying reading tests that are read aloud, we did not use a chin rest, which is normally used for head stabilization in infrared oculography. This meant that we had to recruit very cooperative subjects and only use patients with relatively mild cerebellar ataxia. We had to painstakingly monitor the subjects’ head and body posture throughout the recording and reject recordings that were not of sufficiently high quality.

Our study is also limited by small sample size, lack of inclusion of patients with moderate to severe cerebellar ataxia because of difficulty with infrared oculography, and heterogeneous disease conditions. Another possible limitation of our study is the difference in font size between the number reading task and the word reading task, which was designed on purpose so that the number reading tasks match the original King-Devick test, which is done on a booklet. We believe the difference in font size did not impact reading speed. One study by Rello *et al*. looked at the impact of font size during reading and showed that any font size larger than 18 should be easily read and should not interfere with reading speed [64]. Increasing font size only increases reading speed if font sizes are smaller than 18 [65]. Finally, an important limitation is that we did not control for the impact of cerebellar ataxia on higher order cognitive function in some patients, which may impact visuospatial planning and reading even in single-digit number reading tests. Certainly, word reading may be more affected than number reading by semantic, syntactical, phonological factors, as well as cognitive abilities, language, education, age, attention, and other factors [57, 66–69], so these are important factors to keep in mind in all reading studies of patients with cerebellar ataxia.
